# Empirically evaluating the WHO global code of practice on the international recruitment of health personnel’s impact on four high-income countries four years after adoption

**DOI:** 10.1186/s12992-016-0198-0

**Published:** 2016-10-12

**Authors:** Vivian Tam, Jennifer S. Edge, Steven J. Hoffman

**Affiliations:** 1 Michael G. DeGroote School of Medicine, McMaster University, Hamilton, ON Canada; 2 Cumming School of Medicine, University of Calgary, Calgary, AB Canada; 3 Global Strategy Lab, Centre for Health Law, Policy & Ethics, Faculty of Law, University of Ottawa, 57 Louis Pasteur St, Ottawa, ON K1N 6N5 Canada; 4 Department of Clinical Epidemiology & Biostatistics and McMaster Health Forum, McMaster University, Hamilton, ON Canada; 5 Department of Global Health & Population, Harvard T.H. Chan School of Public Health, Harvard University, Boston, MA USA

**Keywords:** Health worker recruitment, Migration, Health systems sustainability, Impact evaluation, World Health Organization

## Abstract

**Background:**

Shortages of health workers in low-income countries are exacerbated by the international migration of health workers to more affluent countries. This problem is compounded by the active recruitment of health workers by destination countries, particularly Australia, Canada, UK and USA. The World Health Organization (WHO) adopted a voluntary Code of Practice in May 2010 to mitigate tensions between health workers’ right to migrate and the shortage of health workers in source countries. The first empirical impact evaluation of this Code was conducted 11-months after its adoption and demonstrated a lack of impact on health workforce recruitment policy and practice in the short-term. This second empirical impact evaluation was conducted 4-years post-adoption using the same methodology to determine whether there have been any changes in the perceived utility, applicability, and implementation of the Code in the medium-term.

**Methods:**

Forty-four respondents representing government, civil society and the private sector from Australia, Canada, UK and USA completed an email-based survey evaluating their awareness of the Code, perceived impact, changes to policy or recruitment practices resulting from the Code, and the effectiveness of non-binding Codes generally. The same survey instrument from the original study was used to facilitate direct comparability of responses. Key lessons were identified through thematic analysis.

**Results:**

The main findings between the initial impact evaluation and the current one are unchanged. Both sets of key informants reported no significant policy or regulatory changes to health worker recruitment in their countries as a direct result of the Code due to its lack of incentives, institutional mechanisms and interest mobilizers. Participants emphasized the existence of previous bilateral and regional Codes, the WHO Code’s non-binding nature, and the primacy of competing domestic healthcare priorities in explaining this perceived lack of impact.

**Conclusions:**

The Code has probably still not produced the tangible improvements in health worker flows it aspired to achieve. Several actions, including a focus on developing bilateral codes, linking the Code to topical global priorities, and reframing the Code’s purpose to emphasize health system sustainability, are proposed to improve the Code’s uptake and impact.

## Background

There is currently a shortage of approximately 7.2 million healthcare workers worldwide [1]. This global deficit of physicians, nurses, midwives and other skilled health professionals undermines the ability of a healthcare system to adequately treat and prevent disease, and in critical situations, to provide life-saving care for its constituents.

Perhaps unsurprisingly, the regions of the world with the greatest disease burden concurrently experience the greatest health workforce shortages [1, 2]. For example, 19 of the 20 countries with the highest maternal mortality rates are in Africa, and those living with HIV in the continent account for 72 % of AIDS deaths annually. Africa has just 4 % of the world’s health workforce while disproportionately hosting 25 % of the global burden of disease [3, 4]. Further, sub-Saharan Africa suffers a deficit of 2.4 million doctors and nurses, requiring a 130 % expansion of the current the health workforce to close this gap. Comparatively, the continent has 13 health workers per 10 000 population, compared to 68 healthcare workers per 10 000 in the Americas and 110 per 10 000 in Europe [5]. The need for specialist physicians is even more dire, with some countries in sub-Saharan Africa hosting <1 nephrologist per million population [6].

These shortages are exacerbated by the international migration of health personnel, who travel from resource-poor “source” countries to more affluent “destination” countries in search of improved living conditions, increased prospects for and terms of employment, and better opportunities for their families and children [2, 7]. This global migration is enabled by international trade and labour laws, which reaffirm the ability of all health workers to travel in pursuit of a higher standard of living, assuming the destination country is able and willing to accept them [7]. But affluent countries also have a longstanding history of recruiting healthcare workers trained abroad to fill under-staffed areas, especially rural regions and other underserved populations [7]. The range of stakeholders, recruiters, and health professions involved in health worker migration make it difficult to quantify the volume of movement occurring, or to wholly capture the range of policies and mechanisms in place to regulate this phenomenon. However, the added “pull” factor of active recruitment, facilitated by private recruitment agencies, multiple levels of government and NGOs, greatly contributes to the depletion of health workforces in many low- and middle-income countries [1, 2, 7]. It is estimated that Australia, Canada, the United Kingdom (UK) and United States (USA) account for 72 % of foreign-born nurses and 69 % of foreign-born physicians [8]. These countries have collectively saved $4.5 billion USD in education costs by recruiting physicians from countries in sub-Saharan Africa – countries that lose 30 % of their trained health workers annually to medical emigration [8]. Exemplifying the volume of economic losses to source countries, it is estimated that Kenya loses $500 000 USD per emigrating physician in investment, and $300 000 USD per nurse [9].

In Canada alone, 22 % of resident physicians are trained abroad, the majority of which immigrate from Africa, and the number of sub-Saharan Africa trained physicians in Canada has increased by 60 % over the past ten years [9–11]. In order to mitigate the tensions between health workers’ rights to migrate and the critical shortage of health personnel in many source countries, in 2004 the World Health Assembly (WHA) requested that the World Health Organization (WHO) and its member states create a framework to guide the ethical recruitment and migration of health workers [12]. The call for the development of a global code applicable across all countries, health professions and sectors was situated in the context of intensifying calls for action preceded by multiple bilateral and international agreements intended to regulate these migratory patterns (Table 1) [12, 13].

**Table 1:** Comparing the various current international Codes on health workforce recruitment

Code	Stated objectives	Scope	Implementation mechanism	Considerations for developing countries	Distinguishing features
WHO Global Code of Practice on the International Recruitment of Health Personnel (May 2010)	Establish and promote voluntary principles; Serve as a reference to improve legal framework; Provide guidance in the formulation and implementation of bilateral agreements; Facilitate and promote international discussion and cooperation	Global	Bilateral agreements among states and other supplementary international legal instruments	Destination countries should respect the overriding legal obligation of health personnel to fulfill their working obligations in home countries and seek not to recruit them Destination countries should provide financial and technical support to developing source countries	Establishment of national health authority to provide updates on Code implementation and exchange information on health workforce migration to the WHO Secretariat Global scope: considers rights and obligations of both source and destination countries and migrant health personnel
WFPHA Code of Ethics Pertaining to Health Worker Recruitment from Developing Countries (May 2005)	Judiciously manage the employment of health professionals from abroad	International—applies to all member states of the WFPHA	Mandating WFPHA governments work only with employers that comply with the Code	Low-income countries receive something in compensation for sending health professionals (e.g. health worker exchange programs, government remuneration, continuing education for workers)	Builds upon UK DoH Code of Practice by restricting recruitment from developing countries that only have bilateral agreements with WFPHA Proposes definition for “active recruitment”
UK Department of Health Code of Practice for the International Recruitment of Healthcare Professionals (Dec 2004)	Offer principles and best practice benchmarks to be met in order to supply and manage international health professionals in an ethical manner. Provide targeted recruitment guidelines, education and language proficiency requirements, and employment laws related to international recruitment in order to establish ethical practice (DOH, 2004).	Regional – applies to employers of the UK’s National Health System	Mandating NHS to work only with recruitment agencies that comply with the Code	Aims to prevent the active recruitment of healthcare workers from developing countries unless a government-to-government agreement to support recruitment exists Manages migration with respect to active recruitment, but does not advocate for the retention or training of health workers in either the source or destination country	First national code of practice for international recruitment Best practice benchmarks to gauge adherence to core principles Online registry of commercial recruitment agencies complying with the code of practice If non-compliance by an NHS-approved recruitment agency is suspected, a grievance application can be made to the NHS employers; an investigation of the offending agency will be performed and if found guilty, the offending agency will be removed from the approved list and can no longer supply workers to the NHS.
Commonwealth Code of Practice for the International Recruitment of Health Workers (May 2003)	To provide Commonwealth governments with a framework for the ethical international recruitment of health workers to take place, taking into account the impact of such recruitment on source countries	International – applies to all governments of the Commonwealth nations	Promote dialogue among developed and developing countries to resolve this challenge Follow-up with bilateral and other contractual agreements, e.g. bonding health workers	Acknowledges that recruitment diminishes the source country’s human resources and negatively impacts health systems. Bilateral agreements should be drafted to regulate the recruitment process. All employment agencies must be bound by this Code and governments must set up regulatory systems for recruitment agencies and implement mechanisms to detect noncompliance [10].	Proposes its scope go beyond Commonwealth nations and be taken as a proposed global code of practice on this issue

The resulting *WHO Global Code of Practice on the International Recruitment of Health Personnel* is a voluntary, non-binding instrument that was adopted by the then-193 member states at the 63^rd^ WHA in May 2010 [12]. The Code delineates aspirational goals for health workforce development in both source and destination countries: the former are encouraged to achieve workforce self-sufficiency through improved retention of health workers, while the latter are encouraged to refrain from actively recruiting from countries with critical health personnel shortages [12, 13]. The Code also mandates a renewed focus on improving the education, living and working conditions of health workers in source countries to mitigate the “push” factors that encourage emigration (Table 2) [13]. Knowledge- and resource-sharing to strengthen source countries’ health human resource capacity is also emphasized [13].

**Table 2:** Summary of “push” and “pull” factors on the migration of health workers

Push factors encouraging emigration from source countries include:
• Poor remuneration • Concerns for personal safety • Few career prospects and opportunities for promotion • Poor working conditions and heavy workload • Poor living conditions
Pull factors encouraging immigration to destination countries include:
• Better remuneration • Safer environment • Professional development and career advancement opportunities • Improved working conditions and facilities • Higher standards of living

However, as an instrument without clear incentives, institutional mechanisms, or interest mobilizers, the Code may not be able to induce the changes sought, instead making the implementation of its provisions dependent on countries’ own willingness and capacity to act upon the norms it proposes [14, 15]. As such, the success of the Code can be inferred in part from the policy and regulatory changes voluntarily adopted by member states to comply with its provisions. In 2011, Edge & Hoffman (co-authors of this study) conducted the first empirical impact evaluation of the Code to determine its efficacy in affecting national and sub-national change [13]. Their findings suggested that the impact of the Code was limited at eleven-months post-adoption, with a majority of their key informant survey respondents indicating that the Code had had little meaningful impact on policies or regulations governing health personnel recruitment in their countries of work [13]. The authors postulated that the mere adoption of the Code was insufficient to fill the gap between high-level demands for action at the international level and the knowledge and practice of sub-national stakeholders, and called for greater advocacy and awareness of the Code at the national and sub-national levels [13]. The need for increased prioritization of the Code’s principles was emphasized by multiple studies evaluating its impact in the short-term [13, 16, 17].

Reports of country-specific implementation collected by WHO yielded similarly disappointing results. To monitor global progress on the Code’s implementation, each member state was asked to designate a National Authority that would be responsible for reporting on the country’s implementation status every three years using a universal National Reporting Instrument [12]. This instrument is a cross-sectional self-assessment survey assessing progress on all 10 articles of the Code [12]. In the first and only reporting period to date (March-June 2012), less than half of all signatories had formally designated a National Authority (44 %), while only 37 of the possible 193 member states (19 %) reported having taken steps to implement the Code. However, even these numbers may be an overestimate, as the instrument relies solely on self-reported answers to close-ended questions. No supplementary mechanisms exist through which to ensure questions are appropriately interpreted, or to verify the validity of responses, rendering the tool limited in its ability to capture the real situation and nuances of each country’s experience.

In spite of these various challenges, the Code does seem to have helped galvanize international attention for the importance of ethical health personnel recruitment. It received renewed commitment through the Recife Declaration adopted at the Third Global Forum on Human Resources for Health, and has been cited by many countries as an important impetus for action on strengthening their health human resource capacity [16, 18, 19]. However, while it is difficult to isolate the impact of the Code on health worker migration, there certainly have been no notable improvements in health workforce flows from regions of the world where the shortages are most dire: 2013 data from the American Medical Association Physician Masterfile (AMAPHM) demonstrated a 5.4 % annual growth rate in the number of sub-Saharan African-trained physicians joining the US physician workforce, while the proportion of African-trained physicians in Canada has continued to increase from 1996 to date [20–22]. Despite the apparent increasing recognition of the severity of the global health worker shortage, little measurable progress has been made to support implementation of the Code and to affect global regulatory change. The Code’s insufficient uptake was highlighted at the 67th World Health Assembly in May 2014, when WHO was asked to develop a Global Strategy on Human Resources for Health: while the Code was cited as a “watershed” moment for global health human resources, its current utility was said by some commentators to be primarily as “a reporting exercise, rather than a catalyst for improved health workforce policy” [23].

This study represented a medium-term empirical impact evaluation of the Code, four years after its adoption. It sought to determine whether there have been significant changes in stakeholders’ perception of the implementation, utility, and relevance of the Code since the initial evaluation conducted by Edge & Hoffman over three years prior [13]. It also aimed to determine whether the Code’s provisions reached their end-user, namely the policymaker, recruitment official or health workforce regulator, who would be expected to have awareness of or have contributed to changes in policy and practice made in direct response to the Code. Finally, by evaluating the real-world impacts of the Code, this study aimed to help build the evidence base on whether such non-binding agreements are capable of inciting the tangible changes they aspire to.

## Study design and methods

This follow-up study repeated the same methodology that was initially developed for the first empirical impact evaluation of the Code conducted only eleven months after its adoption by the WHA [13]. Specifically, a survey sampling frame of 393 civil society, government and private sector key informants from the four English-speaking countries with the highest volume of migrant health personnel – namely Australia, Canada, UK and USA – was assembled from the participant list of the Third Global Forum on Human Resources for Health and the International Health Workforce Collaborative, through snowball sampling, and from purposive internet searches for authors of relevant publications from each country. The search for these publications was conducted between May and June 2014. Examples of search terms used included “human resources for health”, “international health worker recruitment”, and “health worker migration”, along with one of the four country names. Individuals were selected for their role in directly recruiting, regulating, setting policies about and/or researching the international recruitment and retention of health personnel, based on information from profiles available in the public domain.

The survey instrument from the original study was used to facilitate the direct comparability of responses [13]. Survey questions aimed to evaluate informants’ awareness of the Code and knowledge of any regulations and/or recruitment policies that were changed as a result of the Code’s adoption. The survey also assessed respondents’ perceived impact of the Code and their opinions of non-binding instruments more broadly (Table 3). An initial invitation email was sent to all participants in May 2014, followed by three reminders over June-July 2014. Final responses were received in August 2014 and analyzed thereafter.

**Table 3:** Survey instrument

1. From your perspective, to what extent are your colleagues generally aware of the WHO Global Code of Practice on the International Recruitment of Health Personnel in your field of work (consider knowledge of the Code’s purpose and contents)?
2. Are you aware of any anticipated changes to take place within your country as a result of the WHO Code of Practice (e.g. influence decisions relating to health policy, health professional regulation, health facility administration, recruitment practices)?
3. Do you know of any particular examples where the WHO Code of Practice influenced specific changes? Please describe any examples that come to mind.
4. From your perspective, has the WHO Code of Practice resulted in any changes to the way that health workers are recruited to your country? If so, how are health workers recruited differently? If not, do you know why changes did not take place (e.g. too soon to implement recommendations, processes were already compliant with the Code, changes were made previously in response to other factors, recommendations in the Code were not feasible)?
5. From your perspective, in what ways has your own work changed as a result of the WHO Code of Practice or as a result of policy changes that were made directly due to the WHO Code of Practice? If no changes have occurred so far, do you expect any changes in your personal work will take place in the future?
6. Based on your personal knowledge and experience, please rate your level of agreement with the following statement by marking a “X” beside the numerical value associated with your rating [please mark only one “X”]: “The WHO’s Global Code of Practice on the International Recruitment of Health Personnel has had a meaningful impact on health workforce recruitment, practices, policies, or regulations in my country.”
i. _____Strongly Disagree [1]
ii. _____Moderately Disagree [2]
iii. _____Slightly Disagree [3]
iv. _____Neither Agree nor Disagree [4]
v. _____Slightly Agree [5]
vi. _____Moderately Agree [6]
vii. _____Strongly Agree [7]
7. From your perspective, are there any general or specific changes that could be made to the WHO Code of Practice to improve its impact on the health workforce recruitment, practices, policies, or regulations in your country? Please describe any amendments that come to mind.
8. Are you aware of the complementary guidelines informing the implementation of the Code of Practice? If so, are you aware of any instances when the guidelines have been applied in your work or the work of your colleagues? Please describe any instances that come to mind.
9. From your perspective, do you think these kinds of voluntary, non-binding global codes of practice are effective instruments for influencing change in your country? Please explain why or why not.

### Ethics approval

This study was approved by the McMaster University Research Ethics Board in Hamilton, Ontario, Canada. To facilitate confidentiality, participants were assigned a unique identifier based only on country and sector; identifying details including job titles or affiliated organizations were dissociated from responses prior to analysis.

### Data analysis

A thematic analysis approach was followed to extrapolate key themes from the qualitative responses. This involved an initial sorting of the responses into constructed descriptive categories and the subsequent definition of analytical themes. Themes were thus determined by the data itself and not in accordance with any initial hypotheses that might have influenced results. Quantitative descriptive statistics were extracted based on the coded responses, and representative quotations were identified.

## Results

A total of forty-four responses were received with all four countries and three sectors represented, yielding an approximate response rate of 11 %. Civil society respondents were from academic institutions, think-tanks, research centres, and workforce planning organizations. Government respondents were from workforce regulation agencies and ministries of health. Private sector respondents were from health worker recruitment and training agencies. Respondent job titles included Associate Deputy Minister, Deputy Director, Senior Team Leader, and Senior Health Economist, among others.

### Awareness of the Code

Eighteen respondents (41 %) indicated that they were largely unaware of the Code and its impact despite working in organizations for the licensure of international medical graduates (*n* = 2), health workforce modeling (*n* = 4), health workforce planning and research (*n* = 8), health policy and workforce development (*n* = 3), and international workforce equity (*n* = 1).

Ten respondents indicated that their colleagues lacked an awareness of the Code (23 %). One private sector respondent from Canada stated, “Neither the existence of such a global Code, nor the WHO role is known within our health services network” (CAN.PS.1). A civil society respondent from the USA emphasized, “[My colleagues] are unaware of the problem and any possible solutions” (US.CS.1). The two respondents reporting some level of awareness suggested that the Code was likely more familiar to colleagues in developing countries. One respondent mentioned, “My impression is that my Canadian colleagues have minimal awareness of the Code…while colleagues in developing countries where we work, such as Jamaica and Zambia, are much more aware of it” (CAN.CS.2).

Ten respondents reported that their colleagues were likely to be familiar with the Code (23 %). Of these, colleagues who were researchers and senior academics were reported as likely knowledgeable about the instrument (*n* = 5). However, colleagues whose work directly impacted health workforce planning, such as those working in health authorities and the health policy community were reported to be generally unaware of the Code (*n* = 4).

Among the three individuals reporting that their colleagues were “very aware” of the Code (7 %), two individuals were from Australia, while one Canadian respondent referred to the Code in conjunction with the Melbourne Manifesto, which, they surmised, collectively provided a basis for discussing issues affecting health worker migration. One of the Australian respondents stated, “I believe all senior academics would be aware of the WHO Code, including its global purpose” (AU.G.1).

### Changes resulting from the Code

Of the four individuals reporting anticipated policy or regulatory changes as a result of the Code, a Canadian government respondent declared, “…we are consistently working with colleagues to ensure that our policies follow the Code – I would say that this would be true of all jurisdictions in Canada” (CAN.G.1). However, the vast majority of respondents indicated that they were not aware of any anticipated changes resulting from the Code (*n* = 21; 48 %). One UK respondent suggested that, as the UK Code of Practice on International Recruitment preceded the WHO Code, it was difficult to determine any specific impacts of the latter. Alternatively, a number of respondents attributed poor implementation to the primacy of domestic health workforce needs and competing priorities. One Canadian civil society respondent claimed, “…I expect there to be a greater emphasis on international recruitment of health workers to Canada as both the general population and key health cadres such as nurses continue to age” (CAN.CS.2). The prioritization of national deficits was echoed by American and Australian respondents, one of whom suggested, “My personal commitment is to produce a workforce in and for the regions in which I work. However, I’m heartened that this social justice issue is on the national and international agenda” (AUS.CS.3). A civil society respondent from the USA corroborated, saying “there is very little incentive for healthcare providers to adhere to the Code – there is actually more domestic incentive to *not* adhere to the Code, for example through increased funding for ‘diversity’ hires” (US.CS.5).

Key informants suggested that strong personal incentives for health workers to leave their countries of origin explained the lack of change the Code had hoped to achieve. These “pull” factors included the right of individuals to migrate for improved political and socioeconomic stability, as well as to achieve a higher standard of living (*n* = 3). One Canadian civil society respondent stated, “The real incentive to keep people in their country of origin is addressing the social inequalities in these countries and providing supportive environments…for them to build meaningful lives in safe conditions. People don’t leave unless they really have to” (CAN.CS.3).

Existing national or bilateral agreements were also seen as preeminent to the Code, with respondents referencing tangible regulatory changes following the implementation of the Melbourne Manifesto and the UK Code of Practice, among other international codes (*n* = 4). The Code was often seen as complementary to these existing agreements, or providing further leverage for their support, albeit largely incapable of inciting change directly. As one civil society respondent from the UK mentioned, “…the UK already had a Code of Practice, [but] the WHO Code has proved useful in terms of encouraging cross departmental work in monitoring the UK Code” (UK.CS.1). One American civil society respondent also pointed out the “synergistic” impact of the WHO Code in legitimizing the work of NGOs in trying to reduce unethical international health worker recruitment (US.CS.8).

### Suggestions to improve the Code’s impact

When asked whether the Code had a meaningful impact on health worker recruitment, fifteen respondents disagreed (34 %), with six and eight individuals indicating strong and moderate disagreement, respectively. However, thirteen respondents suggested that no specific amendments to the Code would improve its effectiveness in terms of producing change in health worker recruitment policy or regulation. In explanation, two respondents suggested that the Code’s “principles were good” (CAN.CS.1) and that it was “fairly comprehensive” (US.CS.5), while others cited current domestic changes to reduce health worker recruitment that were independent of efforts to implement the Code (*n* = 2). One American civil society respondent explained, “[The USA government has] strengthened their investments in health professional education domestically. This was not a result of the WHO Code but it has helped alleviate the problem” (US.CS.8).

Of the eleven respondents suggesting possible changes to the Code to strengthen its impact, four suggested the principles of the Code be more widely disseminated, while nine promoted the idea of international public reporting of individual nations’ adherence. As one Canadian civil society respondent pointed out, “I believe the Code itself is a fully developed tool, but regular international public reporting on developed countries with high levels of their health workforce educated in the second or third worlds would track the relevance or effectiveness of the Code” (CAN.CS.6). Five respondents urged clearer requirements for accountability and consequences for non-adherence, with one American civil society respondent favoring a tax for destination countries and corresponding financial compensation for source countries. However, despite supporting improved accountability mechanisms, one Canadian civil society respondent noted a possible barrier to doing so, citing “…those who are reporting are not those recruiting so there is a mismatch between activity and accountability” (CAN.CS.8). Two civil society respondents suggested promoting action on the social determinants of health to improve living conditions, while a UK government respondent urged, “the [Code] needs to be turned into a monitoring board based on indicators…intentions are nice but numbers are more tangible” (UK.G.1).

### Effectiveness of non-binding Codes

Few respondents were of the opinion that voluntary, non-binding codes such as the WHO Code were effective instruments through which to influence national policy (*n* = 3). The majority of respondents indicated that the Code was only “somewhat” effective, with proponents offering the rationale that the Code raised the international profile of the issue (*n* = 4) and was an effective tool to support advocacy efforts or to justify implementation of existing bilateral agreements (*n* = 5). One respondent suggested a particular utility of the Code in enabling the “naming and shaming” of non-adherent countries, claiming, “wealthy countries such as Australia have ‘pride’ and would not wish to be exposed as an exploiter and so have made serious endeavors to adhere to the guidelines” (AUS.CS.3).

Participants reaffirmed that the Code was useful in principle, but that its utility was undermined by national health systems that involved multiple levels of leadership. As stated by one American civil society respondent, “They can be [useful], if they are created with the active participation of the leaders in the field. At least in the USA, that leadership is widely distributed, fluid, and definitely not of a single mind” (US.CS.4). These sentiments were echoed by another American respondent, who argued, “Given that the USA does not have a national health system, disseminating this information and getting multiple different systems and entities to follow this, I feel, will be virtually impossible” (US.CS.3).

Ten participants asserted that voluntary, non-binding Codes were not effective. Respondents cited a lack of necessary accountability mechanisms (*n* = 5), in addition to prevailing health inequities that remain the primary incentive to migrate (*n* = 3). Internal pressures to fill under-resourced health sectors, and a sense that the Code was more highly esteemed by developing countries were also listed as reasons for its ineffectiveness. One Canadian participant explained, “In developed countries, there is little sense that these types of WHO edicts apply to them, whereas in low-income countries, the WHO carries a lot of weight. In this case, the voluntary, non-binding nature of the Code makes it a low priority for all” (CAN.CS.5).

## Discussion

### Comparative principal findings between current and initial study

The main findings between the initial impact evaluation of the Code and the current one (conducted at eleven months and four years post-adoption, respectively) are strikingly similar. In both studies, the majority of key informants reported that no significant policy or regulatory changes to health worker recruitment had occurred in their countries as a direct result of the Code. To explain this lack of impact four years after adoption, we turned to global health governance frameworks developed by Hoffman and Røttingen (2015) and Happaerts (2012) whose shared principles provide a theoretical basis in which much of the analysis of our responses can be grounded [14, 24].

In assessing the effectiveness of global health treaties, Hoffman and Røttingen (2015) derived three characteristics necessary for such agreements to achieve intended social impacts (defined as those without principally economic aims). These included: 1) incentives for participating actors in alignment with their self-interest and priorities 2) institutional mechanisms to promote compliance or penalize divergence from treaty principles and 3) interest mobilizers, or strong coalitions and advocacy groups with sufficient resources to promote implementation [14]. Similarly, Happaert (2012) posits that successful uptake of ‘outside-in’ policies (those that are internationally defined but rely on national and sub-national leadership for implementation, such as the Code) requires both 1) the compliance of national governments with binding agreements, or ‘international harmonization’ and 2) the promotion of policy models, or ‘norm-setting’ by international organizations. [24] The latter is further explained by Bernstein and Cashore’s discussion of international normative discourse (2002), where international organizations like the WHO are able to shape domestic policy by first defining principles of appropriate behaviour and then driving their international institutionalization by developing a pervasive discourse around them (through mechanisms such as global fora and inter-state conventions [24].

### Bilateral or regional Codes in incentivizing compliance

To explain the Code’s lack of perceived uptake, many participants emphasized a lack of incentives for compliance (due to its voluntary, non-binding nature) as well as the existence of disincentives for adherence (including the primacy of fulfilling domestic health worker needs. In both studies, the Code’s impact was perceived to be secondary to that of the multiple existing bilateral or region-specific Codes. The precise nature of these agreements better approximate each party’s self-interest (thereby providing adequate incentive), and include institutional mechanisms such as penalties for non-compliance, thus also facilitating international policy harmonization. For example, during the 1990s the proportion of foreign-trained physicians working in the UK had increased steadily as a result of recruitment efforts directed at low-income countries [25–29]. In 2001, the UK Department of Health introduced the UK’s Code of Practice on International Recruitment to mandate that no National Health Service (NHS) employers would engage in active recruitment of health workers from low-income countries in recognition of its detrimental impacts on the latter’s health workforce capacity [27–29]. Penalties for offending agencies include revocation of ability to supply health workers to the NHS, which was particularly significant given that the NHS remains the UK’s largest employer and most important deliverer of healthcare [27]. An evaluation of the UK Code by Buchan et al. (2009) demonstrated a “considerable” reduction in the influx of health workers to the UK following implementation of the Code: by 2007, only 45.2 % of all new physicians were international medical graduates, compared to 74.7 % in 2003, with a concurrent decrease in the proportion of international medical graduates from low income countries as well (although it was aptly noted that these findings could not be attributed solely, or perhaps even principally to the Code subsequent to the broad range of factors affecting health worker migration) [29–31].

Bilateral agreements also facilitate the development of explicit incentive-institution mechanisms that are agreeable to both nations and thus enable policy harmonization. For example, the Philippines had become the lead source country of nurses worldwide, and had the largest proportion of registered nurses (RNs) working in foreign countries, comprising one-third of all migrants leaving the country with Canada as one of the main destination countries [32–36].

Conversely, in 2008, 50 % of Canadian RNs were expected to retire within 15 years, contributing significantly to the nursing shortage expected: the healthcare needs of its aging population were expected to demand 361 000 RNs by 2016 in the context of a projected shortage expected to reach 113 000 by the same year [37]. The development of the Philippines-Canada memorandum (2008) facilitated regulated labour movement between certain Canadian provinces and the Philippines on the terms that Canada committed to supporting human resources development in the source country, including specifically by describing Canadian funding mechanisms for recruitment [38]. These explicit provisions thus enabled both the source and destination country to achieve better regulation of health workforce flows amongst them, while being able to negotiate terms acceptable to their self-interest.

Similarly, China and the UK entered into a memorandum of understanding regarding health workforce flows in 2006 with unique criteria negotiated to optimize the UK’s health workforce shortages while preventing further health workforce inequalities between urban and rural China. Since 1998, China has produced an excess of health workers relative to their capacity for absorption into the health workforce [39]. Comparatively, the UK had 57 000 fewer nurses than required to fulfill staffing requirements of the NHS in 2001, with already a large proportion of the health workforce coming from low-income countries overseas [26, 28, 29, 40]. However, Chinese physician-density in urban areas was and continues to be twice that in rural areas, whereas nurse-density is tripled in urban vs. rural centres, resulting in within-province inequalities in health worker density of up to 82 % [41]. This rural to urban displacement combined with overall excess health worker production results in a unique set of challenges that China attempted to mitigate in part through bilateral agreements regulating where destination countries including the UK could recruit workers from, resulting in a UK-China memorandum (2006) that mandated that the UK recruit from China through regulated agencies only, and prohibiting any direct recruiting of physicians or nurses and any recruiting from rural areas [38].

Consequently, country-/region-specific codes such as the bilateral agreements and the UK Code have been comparatively more successful in managing health workforce flows than the WHO Code itself, and indeed are a precedent encouraged by the WHO Code. Thus, a focus on developing such targeted agreements would facilitate nations’ compliance with the international norm, while doing so in a way that may more effectively achieve desired outcomes as a result of their binding nature and clearer incentives for adherence.

### Institutional mechanisms for compliance and accountability

The impact of a lack of incentives on the Code’s effectiveness is compounded by a lack of ‘institutional mechanisms’, including penalties to discourage non-compliance or strict accountability mechanisms to monitor progress. Many participants proposed a greater role for the WHO in imposing consequences on deviant countries to better facilitate compliance: participants touted the benefits of levying a “solidarity tax” on recipient countries for each health worker gained, or through “naming and shaming” non-compliant countries through public fora. While substantive penalties are absent from the voluntary Code by definition, the WHO could remedy this deficiency by instead increasing accountability and reinforcing responsibility of destination countries through a renewed focus on gathering more specific, measurable indicators. These could include assessing mechanisms used to increase the size of the domestic workforce, including increasing health professional school enrollment, subsidies for health professional students, and incentives for rural and remote practice. These additional indicators could be used to quantify the health human resources gap more precisely, while also serving as benchmarks of national progress in achieving health workforce sustainability.

### Shaping normative discourse

As a voluntary instrument ratified by the WHO, where the Code was poised and indeed perceived to have the most impact is through shaping the normative discourse around the international health workforce [24]. Indeed, despite the majority of participants believing the Code had not produced tangible changes, a large number of participants reported also their belief that no revisions could be made to improve its impact. Where the first study ascribed the resulting lack of impact in part to insufficient elapsed time, publicity, and support for implementation, little has changed three years later in the way of the Code’s promotion and subsequent uptake. And when the WHO was asked to develop a Global Strategy on Human Resources for Health, the WHO Code was seen more prominently as a watershed moment than as a catalyst for change in global health human resources [18]. Yet participants in this study still described the Code as a “fully developed tool” with sound principles, despite few member states adhering to its norms in practice. Clearly, despite having few implementation or accountability mechanisms, voluntary instruments such as the Code can serve an important role in galvanizing stakeholder attention and subsequently ensuring the issue is prioritized on both global and national agendas [24, 42]. The ability of non-binding codes to raise an issue’s global profile was recognized even by the respondents who suggested that voluntary codes were generally of low- to mixed-effectiveness. These merits of the WHO Code became evident when it received renewed commitment from attendees of the Third Global Forum on Human Resources for Health, which cited the Code as an important global commitment whose principles should be adopted by all countries [18].

### WHO as interest mobilizer

Instead of focusing on the need for external advocacy efforts, the WHO’s inherent capacity as an international norm-setting agency can be capitalized on to serve as its own ‘interest mobilizer’. For example, the Third Global Forum framed the Code and its focus on human resources for health as an integral part of achieving universal health coverage (UHC), which has emerged as a topical global priority [18]. By purposefully linking the Code with current global health priorities, the Third Global Forum highlighted an opportunity for renewing attention to the principles of the Code and encouraging its implementation without expending additional resources on its advertisement. WHO could further capitalize on this momentum by linking the Code with other priorities, such as the Sustainable Development Goals (SDGs), accomplished by referring to the Code in further discussions on UHC and the SDGs, or in global fora or technical consultations concerning the ongoing development of the WHO’s global strategy on human resources for health, most recently presented at the 69^th^ World Health Assembly for draft consideration [43]. These efforts could serve the dual purpose of ensuring that the Code continues to be publicized among member states, and that national and sub-national discussions concerning UHC, the SDGs and Human Resources for Health would inevitably involve a deliberate focus on the state of implementation of the Code.

### Framing and naming to optimize relevance

Finally, eighteen respondents (41 %) indicated that they were unable to provide answers to all questions in the survey, with many suggesting either that their work did not directly overlap with the Code, or that they had limited knowledge of the instrument and its impact. As mentioned, this is despite their working in organizations for the licensure of international medical graduates, health workforce modeling, health workforce planning, health workforce development, and international workforce equity. Many respondents also discussed the idea that the Code was limited in its scope, and that it neglected to address broader drivers of health worker emigration such as poor working conditions or uncertain employment prospects in home countries. However, the Code actually devotes multiple articles to outlining principles of sustainable health workforce development (Table 4). These and other provisions of the Code render it wider in scope and thus more broadly applicable than respondents initially perceived. This is perhaps due in part to the Code’s title, which is narrowly focused on international ethical recruitment. To more accurately capture the Code’s holistic principles, a reframing of its intent and purpose would be prudent. In practice, this can be accomplished by talking about the Code as framework for *health workforce sustainability* – which, in fact, more accurately depicts its purpose and might better attract the attention of stakeholders it was intended to influence. In the future, the naming of global governance instruments should be critically scrutinized to avoid any possible narrowing or misinterpretations of purpose, and to ensure the full range of relevant stakeholders are engaged from the beginning (Table 5).

**Table 4:** Articles of the Code supporting sustainable health workforce development

Sub-articles of the Code encourage member states to:
• Consider adopting measures to address the geographical maldistribution of health workers and to support their retention in underserved areas, such as through the application of education measures, financial incentives, regulatory measures, social and professional support (Article 5.7)
• Consider strengthening educational institutions to scale up the training of health personnel and developing innovative curricula to address current health needs (Article 5.5)
• Include [in bilateral/regional agreements] the provision of technical assistance, support for health personnel retention, support for training in source countries, twinning of health facilities, support for capacity building in the development of appropriate regulatory frameworks (Article 5.2) [7]

**Table 5:** Summary of key policy recommendations

1. Subnational and national stakeholders:
a. Develop bilateral and national Codes of Practice adherent to the norms set by the Code
2. Global stakeholders:
a. Collect data on measurable indicators destination countries are using to increase the size of their domestic workforce
b. Further link the Code and its principles to the achievement of topical global health priorities through press releases, impending forums or technical briefings
3. All stakeholders: Reframe the Code when referenced in national and sub-national fora to reflect its consideration of broader factors contributing to health worker migration

## Strengths and limitations of the study

The main limitations of this study include its relatively small sample size and the distribution of respondents across countries and sectors. This limits the generalizability of the results and introduces an unquantifiable amount of participation bias in the sample. Respondent composition also favored Canadians and civil society, while the private sector and UK were least represented. However, the survey was not intended to be nationally representative, and these limitations are offset by the variety of eminent positions held by the respondents, as well as the high degree of inter-respondent agreement that transcended both sectors and sampled countries. Consequently, the reported results may be indicative of the perspectives of seasoned experts and leaders with broad experience in national and global health human resources.

Additionally, participants were likely self-selective in favour of those who were aware of the Code, implying that many respondents who declined participation were likely unaware of the framework. Thus, the proportion of national and sub-national actors aware of the Code in the four countries could be over-represented in our sample, such that the perceived impact of the Code may be even more unsatisfactory than presented here.

This study has four notable strengths. The first is the timeframe in which it was completed: the WHO had released complementary implementation guidelines for the Code four years prior, and the first national reporting deadline on implementation of the Code had passed (March-June 2012) with significant time elapsed for analysis and reporting. This study was able to build on the analyses provided by the report on country’s implementation as well as other recent analyses of the Code’s effectiveness, and is the first evaluation of the Code to compare empirical impact with that reported by countries. There was also a high degree of concordance between the responses received across sectors and countries, which suggest possibly that an increased number of respondents would likely have yielded similar qualitative results. Many respondents also qualified their responses with rich detail, enabling informed, comparative analyses. Finally, the senior positions held by the majority of respondents qualified them to speak knowledgeably about the perceived impact (or lack thereof) of the Code on their practice.

## Conclusions

The Code was intended to serve as the first globally applicable framework to mitigate the impact of health workforce migration on countries experiencing severe health worker shortages. It was adopted amidst growing international disquiet concerning the high volume of migratory flows from low- to high-income countries, and the global community was lauded for taking a step forward to mitigate this disparity. However, multiple assessments of its effectiveness have now demonstrated insufficient national uptake and implementation of the Code’s principles. Little has changed since the initial impact evaluation of the Code three years ago; since then, the Code has still not produced the tangible improvements in health worker flows it aspired to achieve and WHO has yet to modify its approach on the issue. The deficit of action should raise questions about the potential impact of developing similar global instruments on different health issues in the future. Such codes of practice, in line with the WHO’s capabilities, are intended to set norms, propose policies and encourage institutional arrangements that comply with globally defined standards. Yet this study and the preceding impact evaluation emphasize the bilateral and regional Codes and the importance of sustained promotion and advocacy efforts to support the uptake of voluntary codes’ norms. As the latter has so far been insufficient to support widespread uptake of the WHO Code, further study would be useful to analyze the aggregate impact and relative utility of bilateral and regional Codes in comparison to global instruments looking to achieve similar goals. Perhaps this analysis might yield valuable insight into circumstances of adoption, monitoring, incentives and dissemination methods that can be adapted to optimize the effectiveness of voluntary global codes in the future.

Nonetheless, there may be numerous interventions that could strengthen the Code’s impact. This study highlights several such opportunities that could be pursued by WHO and other global health organizations, such as further linking the Code to top global priorities, collecting data on indicators used by source and destination countries to increase the size of their domestic health workforce, and reframing the Code’s purpose to emphasize health system sustainability to engage a broader set of stakeholders. This set of actions could address the challenges reported by the sub-national stakeholders that comprised the majority of respondents in this study. In the meantime, the Code has drawn global attention to the health workforce crisis, if only at one moment in time. If such codes continue to be developed, the challenge moving forward will be learning how to capitalize on their norm-setting capacity to translate initial motivation and commitment into sustained action.

## Abbreviations

AIDS: Acquired Immunodeficiency Syndrome
AMAPHM: American Medical Association Physician Masterfile
HIV: Human Immunodeficiency Virus
NGO: Non-governmental organization
NHS: National Health Service
RN: Registered nurse
SDG: Sustainable Development Goal
UK: United Kingdom
USA: United States of America
USD: US dollar
WHA: World Health Assembly
WHO: World Health Organization
